# Defect Detection and Imaging in Composite Structures Using Magnetostrictive Patch Transducers

**DOI:** 10.3390/s23020600

**Published:** 2023-01-05

**Authors:** Akram Zitoun, Steven Dixon, Mihalis Kazilas, David Hutchins

**Affiliations:** 1 Brunel Composites Centre, College of Engineering, Design and Physical Sciences, Brunel University London, London UB8 3PH, UK; 2 Department of Physics, The University of Warwick, Coventry CV4 7AL, UK; 3 School of Engineering, The University of Warwick, Coventry CV4 7AL, UK

**Keywords:** guided waves, magnetostriction, SAFT imaging technique

## Abstract

The use of thin magnetostrictive patches to generate and detect guided waves within the composite samples is investigated for defect detection. This approach has been implemented using SH0 shear horizontal guided waves in both CFRP and GFRP plates. A magnetostrictive patch transducer was able to generate SH0 waves with known directional characteristics. The synthetic aperture focusing technique (SAFT) was then used to reconstruct images of defects using multiple transmission and detection locations. The results for imaging defects in both types of material are presented.

## 1. Introduction

Guided waves are elastic waves propagating parallel to the sample surface within structures such as pipes or plates. Examples include the symmetric (S) and antisymmetric (A) Lamb wave modes and shear horizontal (SH) modes in flat plates. These wave modes have been used extensively for non-destructive testing (NDT) and structural health monitoring (SHM) applications, with the aim of detecting structural damage [1].

There can be advantages in the use of guided waves for NDT over conventional bulk mode signals, including a reduced need for scanning and faster inspection. Bulk wave NDT requires the defects to be under or close to the transducer [2,3], whereas guided waves can detect defects tens of meters away from the location of the transducers, although usually with much lower resolution and sensitivity. In addition, the use of bulk waves methods can be challenging with thin structures, as the frequency required to generate waves with a wavelength shorter than the sample thickness can be prohibitively high, and the signal interpretation can be complicated by the reflections generated from the defect interfering with the reflections from the boundaries of the structure [4].

Despite the advantages of using guided waves for NDT or SHM applications, the use of this technology is still difficult and challenging due to wave dispersion and the fact that multiple modes can propagate simultaneously [5]. The SH0 guided wave mode is non-dispersive in flat plates, so the velocity of propagation is independent of frequency, making it convenient for some guided wave inspections. Lamb waves can also be used, where specific modes can be selected by carefully controlling the frequency and wavelength [6].

Piezoelectric transducer arrays are commonly used to generate guided waves [7,8], although electromagnetic acoustic transducers (EMATs) can also be used to generate guided waves using the Lorentz force transduction mechanism [9,10,11], where the operating characteristics can be controlled via the spacing and direction of the Lorentz forces [12]. Guided wave transduction can also be achieved using magnetostriction [13,14,15]. Laser-based ultrasonic systems and air-coupled ultrasonic techniques have also been used to generate and detect guided waves for inspection purposes [16,17,18], but these are often more difficult to operate in a practical setting.

The use of the more common piezoelectric transducer arrays requires firm clamping of each element to the sample surface and often has complex cabling and electrical driving requirements. The use of EMATs placed directly onto the sample surface is an alternative approach, but their operation is limited to conductive materials. Electromagnetically excited magnetostrictive thin film patches [16,17] can be used to generate guided waves on both conductive and insulating materials. The generation and detection of guided waves using such a magnetostrictive patch require the use of an excitation and detection coil and a permanent magnet to bias the patch or magnetization of the patch prior to use, all of which are needed for the creation of both dynamic and static bias magnetic fields. Different configurations are possible for both of these fields [18]. The introduction of a conductive thin film then facilitates both Lorentz forces and magnetostriction to be used to generate elastic waves. Various authors have investigated ways of enhancing the efficiency of magnetostriction in this application, involving, for example, magnetic circuit optimization [19], the use of periodic permanent magnets [20], the enhancement of longitudinal guided waves [21], and the use of materials exhibiting giant magnetostrictive properties [22].

This paper describes the process whereby the choice of particular experimental dynamic and static magnetic field configurations can be used with a magnetostrictive thin-film patch to optimize the generation of particular guided wave modes in composite plates. This can be achieved via either Lorentz forces, magnetostriction, or, indeed, by both simultaneously. It is, therefore, important to study the effects of different geometries of coils and permanent magnets on wave generation. This allows one to optimize inspection by favorably selecting the dominant guided wave mode for a particular experimental configuration, and once optimized, this could then be used to enhance defect detection.

## 2. Choice of Magnet and Coil Configurations

**Figure 1:**
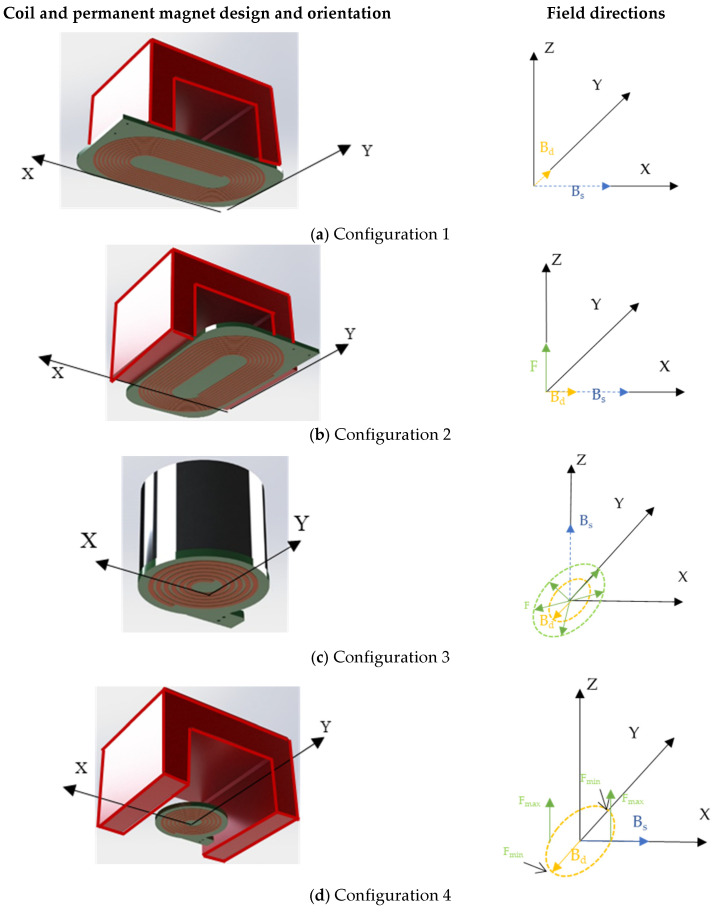
Illustration of the different configurations showing the relative positions of the coil and the permanent magnets along with the relative direction of the static magnetic field of *B_s_* (blue), the dynamic magnetic field *B_d_* (yellow) and Lorentz forces when generated (green).

Here, the task was to investigate the experimental conditions for optimizing a particular guided wave mode when using a magnetostrictive patch. Four different configurations were examined for the case of a magnetostrictive patch attached to a composite plate. The dynamic magnetic field *B_d_* was generated using two main coil designs; racetrack and circular coils were used together with two permanent magnet geometries for providing the static magnetic field *B_s_*. Figure 1 shows the different configurations obtained by combining different coil designs and permanent magnets. In Configuration 1, the racetrack coil (Figure 1a) was used in conjunction with a U-shaped permanent magnet so that *B_s_* was generated in the orthogonal direction to that of the dynamic magnetic field *B_d_*, both lying in the same plane. This was achieved using a U-shaped alnico grade 5 permanent magnet above the linear section of the racetrack coil. In Configuration 2 (Figure 1b), the racetrack coil was rotated by 90° so that *B_s_* and *B_d_* were now in-plane and parallel to each other. In Configuration 3 (Figure 1c), *B_s_* was generated in the out-of-plane direction using a cylindrical permanent magnet, with a circular pancake coil generating an omnidirectional *B_d_* in the X-Y plane (i.e., parallel to the surface of the magnetostrictive patch and sample surface). Configuration 4 (Figure 1d) used the U-shaped permanent magnet in conjunction with the circular pancake coil, which led to the in-plane generation of both a linear *B_s_* and a radial *B_d_* field. The directions of *B_s_* and *B_d_* for the four configurations are shown in Table 1, where the X-Y plane is parallel to the surface of the magnetostrictive patch, and the Z direction is perpendicular to it.

Previous work by the authors has established some of the operating conditions that result from these configurations [23,24], including the radiation patterns of the A0, S0 and SH0 guided wave modes. In Configuration 1, no significant Lorentz forces were expected, leaving only magnetostrictive forces as the driving mechanism. Configuration 1 led to a complex generation pattern for all three modes, but with A0 generation at specific angles. Configuration 2 favored S0 and SH0 generation with in-plane Lorentz forces being present. Configuration 3 allowed both Lorentz and magnetostrictive forces to be generated parallel to the surface of the patch, leading to a quasi-omnidirectional generation of A0, S0, and SH0 modes. Configuration 4 caused the SH0 wave mode to be generated in a particular direction while minimizing the presence of the A0 and S0 modes. These initial tests indicated that variations in *B_s_* and *B_d_* could lead to the generation and enhancement of specific guided wave modes while suppressing others. Further work conducted by the authors [24] investigated the effect of variation of further parameters, such as the excitation frequency and the combined variation of *B_s_* and *B_d_*. It was demonstrated that the magnetostrictive patch provided a wide active transduction range, but that sensitivity was optimum at an excitation frequency of 120 kHz. This frequency was thus selected in order to provide a good sensitivity to signal detection while retaining guided wave signals suitable for the type of defects to be investigated.

Note that the current through the coils was relatively low (<100 A), and thus we expect any self-field Lorentz generation mechanism from the coil alone to be insignificant. Lorentz forces are expected to be minimal in Configuration 1, and that is due to the fact that eddy currents generated from the alternating current and the static magnetic field are generated in the same direction. Lorentz forces are generated in the out-of-plane direction in Configuration 2 and in the in-plane radial direction in Configuration 3. In Configuration 4, Lorentz forces are generated in the out-plane direction with a complex distribution of Lorentz forces. These Lorentz forces are in addition to those arising from magnetostriction in the patch.

**Figure 2:**
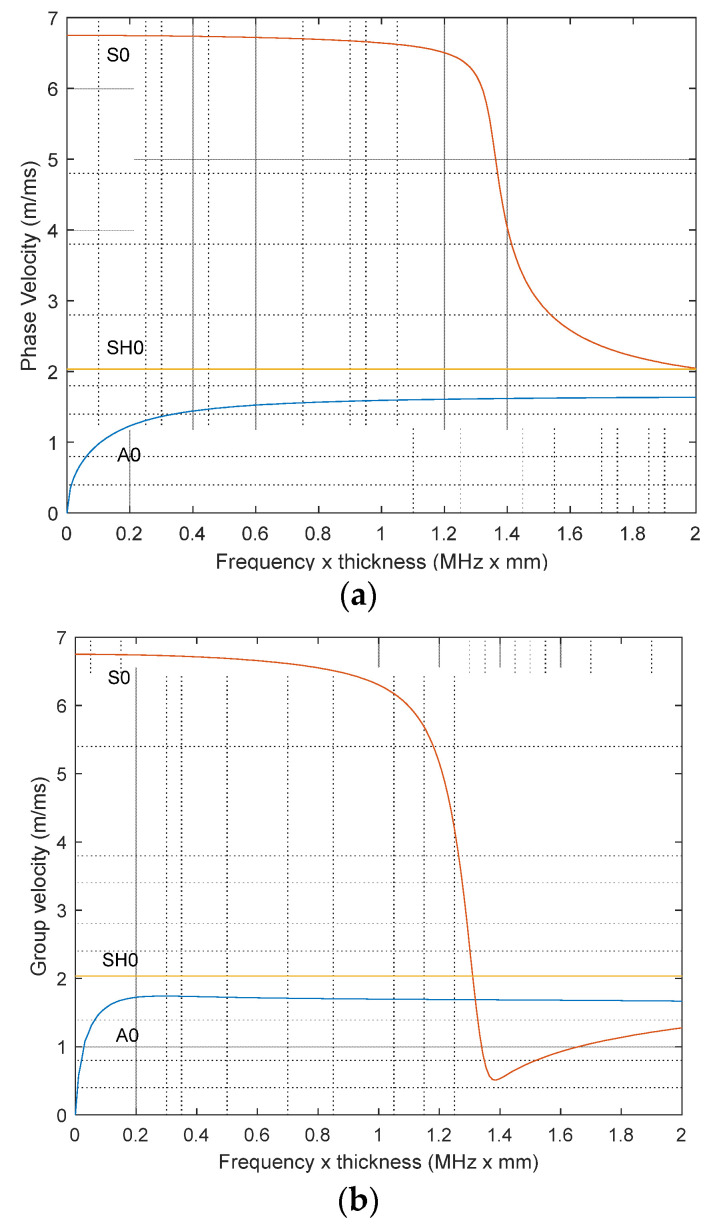
Computed (**a**) phase and (**b**) group velocity for S0, A0, and SH0 wave modes generated in a 3 mm CFRP plate.

As will be described below, the experiments were performed with the magnetostrictive patch attached to a 3-mm-thick CFRP plate made of 16 plies stacked in [0° 90° 0° 90°] lay-up design. It is important to establish the dispersion curves of the different modes that would be expected in this type of sample. These have been modeled, and the results are shown in Figure 2 for both phase and group velocity.

The computed velocities for each of the three modes in the CFRP plate are summarized in Table 2 for an operating frequency of 120 kHz. This allowed the arrival time of a particular mode to be determined in cases where more than one mode is generated by a particular configuration. In particular, it allows the distinction between the arrival time of the shear horizontal wave mode SH0 and the antisymmetric A0 Lamb wave mode, as these are likely to be the dominant modes in the experimental signal.

## 3. Experimental Apparatus

The racetrack coil used in Configurations 1 and 2 (Figure 1a,b, respectively) was constructed using a 1 mm wide copper track with 10 turns. Each turn was spaced by 0.5 mm. The copper track was applied to a printed-circuit FP4 epoxy substrate with a thickness of 35 µm. The overall copper track was 14.5 mm wide, and the full thickness of the printed circuit board (PCB), including the coil, was 1.57 mm. The permanent magnet was placed on top of the linear section of the racetrack coil. In Configurations 3 and 4, the circular coil design was used in conjunction with a circular and U-shaped permanent magnet, respectively. The circular coil was formed of five turns of copper track of 1 mm width at a 0.5 mm spacing.

The U-shaped permanent magnet was an alnico grade 5 magnet with a remanent magnetic field of 1.1 T. The generated static magnetic field, as measured using a gaussmeter (Hirst GM08), was 210 mT at the center of the magnet pole. Note that the actual magnetic field generated when the magnetostrictive patch is present will be higher, as the patch will have higher permeability levels. The remanent magnetic field of the cylindrical magnet used for Configuration 3 was 1.28 T, and the actual generated static magnetic field measured using the GM08 gaussmeter was 300 mT. The magnetostrictive patch was attached to a 3-mm-thick 400 mm × 400 mm CFRP plate using double-sided adhesive tape. This meant that the patches were easily removable for this NDT technique. The selection of the permanent magnets was performed in order to ensure that the operational point of the magnetostrictive patch, when subject to the fields, was at the linear section of the corresponding magnetostrictive curve. In fact, when operating within the linear gradient of the magnetostrictive curve, higher magnetostrictive ratios can be obtained with small changes in the magnetic field from the coil.

The magnetostrictive patch was a 400-mm-long strip patch of 40 mm width. This allowed a transmitter/receiver pair of a particular coil/magnet configuration (Figure 1) to be scanned along the strip for imaging purposes. The magnetostrictive material was an iron–cobalt alloy (FeCo), in this case, a 55-µm-thick VACOFLUX 48 alloy (^®^VACUUMSCHMELZE GmbH & Co, Hanau, Germany). VACOFLUX has a high permeability which can reach up to 18,000 NA^−2^. The mechanical and magnetic properties of the patch are presented in Table 3. The composite plate to which the patch was attached was fabricated with 13 plies in a [0° 90° 0° 90°] layup configuration. In order to investigate the defect detection capabilities, a 20 mm diameter circular defect was machined into the plate to a depth of 1.5 mm.

**Figure 3:**
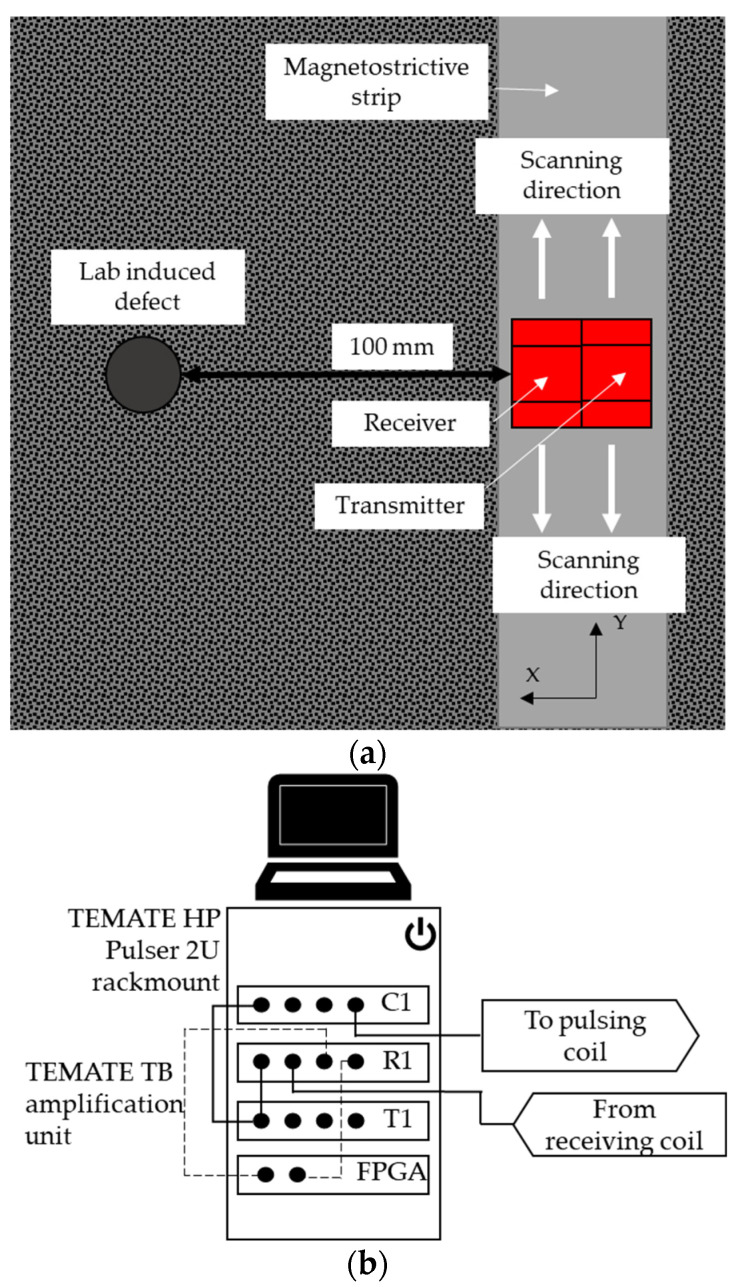
(**a**) Schematic diagram of the experimental arrangement on the CFRP plate containing a circular defect, showing the magnetostrictive strip along which the transmitter/receiver pair was scanned. (**b**) The apparatus used for generating and receiving signals.

To perform the inspection of the composite plate, the transmitter was placed behind the receiver, across the thickness of the magnetostrictive strip, and both were then scanned in unison laterally along the strip in 1-mm steps so as to collect data at regular spatial intervals for later image reconstruction (see Figure 3a).

The coil used to generate the dynamic magnetic field *B_d_* was connected to a custom-designed and manufactured Innerspec RF current pulsing unit supplied to our specifications. It included a TEMATE HP Pulser 2U rackmount, combined with TEMATE TB amplification unit delivering up to 2 KW per channel. The coil embedded within the receiver was connected to the DAQ of the same system, as shown in Figure 3b. The triggering for the DAQ was performed internally within the Innerspec system. The sampling rate of the detector was 25.6 MHz, and the dynamic magnetic field coil was subject to a nominal current power amplitude of 1.8 kW. The built-in FPGA card was used to design a digital filer to apply a smoothing function of 32 points and also to establish multiple electronic gates. These gates were programmed to track different features within the received ultrasonic waveforms to distinguish between different mode arrivals. These gates assisted in identifying the reflections of the backwall, edges and the defect within the CFRP plate.

**Figure 4:**
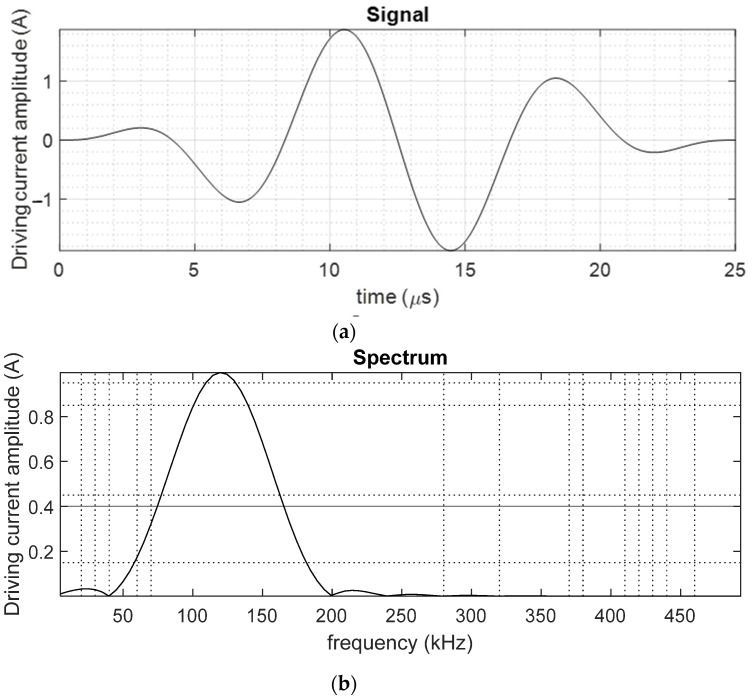
(**a**) Current waveform induced in the excitation coil connected to the RF pulsing unit within the Innerspec system and (**b**) its frequency spectrum.

The current used to drive the coil to generate *B_d_* was a three-cycle gated sine-wave signal at a center frequency of 120 kHz, as shown in Figure 4, together with its frequency spectrum.

Further experiments were also performed on a 1 m × 1 m square GFRP plate of 4 mm thickness. This contained an artificial defect in the form of a 15 mm × 25 mm rectangular PTFE layer, introduced into the GFRP plate during manufacture at a 2 mm depth.

## 4. Results

### 4.1. Effect of Different Magnetic Configurations on the Detection of the Defect in the CFRP Plate

The results are first shown for the detection of this defect using either the SH0 mode or the A0 mode. As expected from the discussion above, the S0 mode signal was of much lower amplitude than that of the SH0 and A0 modes, and hence the S0 mode was not used for defect detection.

**Figure 5:**
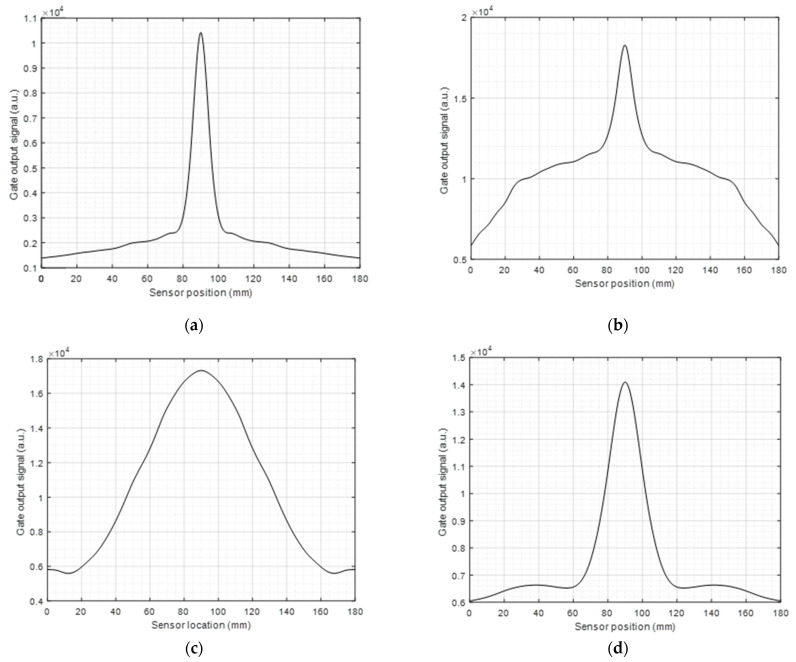
(**a**–**d**) Defect scanning and detection using a 1 mm linear sweep for Configurations 1–4, respectively when using the SH0 mode.

Figure 5 presents the amplitude of the reflected SH0 signal from the defect for all four configurations as a function of the lateral position of the scanned transmitter/receiver pair along the magnetostrictive strip (Figure 3a), over a distance of 0–180 mm in 1 mm steps. The defect was located at the 90 mm position along the scan (i.e., at the center of the scan). Configuration 1 returned a good indication of the defect, mainly because it had a highly directional SH0 beam (see [19] for more details). Although Configuration 2 also generated the SH0 mode, the detection aperture was wider when compared to that of Configuration 1. Configuration 3 returned the widest aperture of defect detection due mainly to its omnidirectional generation characteristics. Configuration 4 provided a similar pattern to configuration 1 in terms of detection capabilities but was slightly wider. These apertures can be used when designing the sensor to provide the desired detection capabilities.

**Figure 6:**
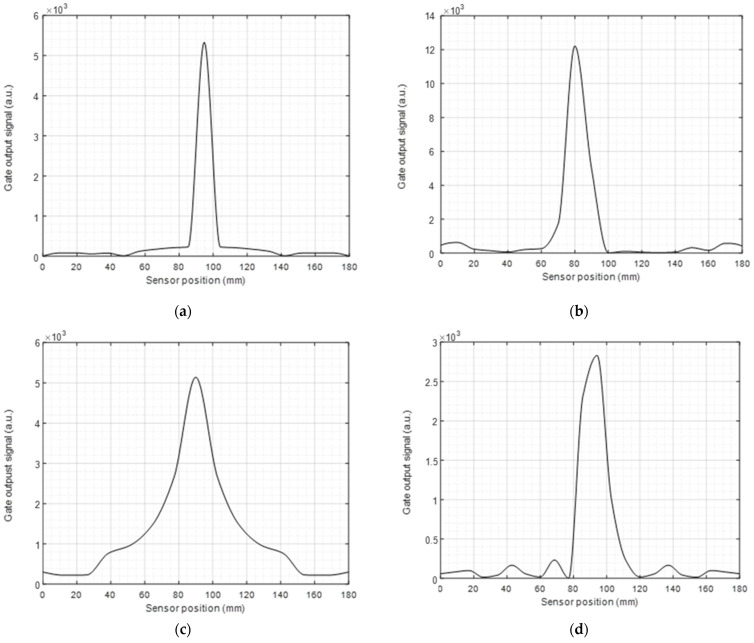
(**a**–**d**) Defect scanning and detection using a 1 mm linear sweep for configurations 1–4, respectively when using the A0 mode.

A similar approach was used for measurements using the A0 mode, and the results are presented in Figure 6. It can be seen that, as for the SH0 mode, Configuration 1 returned the best detection characteristics because of the good directivity. Configuration 2 was able to detect the defect but exhibited a long “tail” on either side of the main defect location, thought to be a result of the less directional radiation pattern. Configuration 3 again exhibited the widest detection aperture because of its omnidirectional nature. Finally, Configuration 4 provided a reasonable detection capability but with a wider aperture than Configurations 1 and 2.

These initial results demonstrated that the magnetostrictive patch approach was able to provide good flexibility in terms of defect detection, in that both the A0 and SH0 guided waves modes could be used to detect the defect, but with different characteristics in terms of directivity and hence aperture size for imaging for each configuration.

### 4.2. Use of the SAFT Technique for Imaging the Artificial Cirular Defect in a CFRP Plate

The synthetic aperture focusing technique (SAFT) is a well-established technique used to reconstruct an image from collected reflected signals using ultrasonic waves [25,26]. Initially, the data were collected as a series of A-scans, and then different algorithms were used to reconstruct the image in either the time or frequency domain. Phase Shifting Mitigation (PSM) is a well-known algorithm used as part of SAFT in the frequency domain [27,28]. PSM was implemented by performing a linear sweep of a sensor across the sample, where a Fourier transformation and phase shift factor were subsequently applied to the received signal. Finally, the results were inverted back to the time domain. A waveform containing 64,000 data points was collected at each spatial step of 1 mm. The sensor and received were both operated manually, assisted with an automated arm. In fact, in order to reduce the overall error and reading errors, the robotic arm with a ruler was used to ensure that the step was equal to 1 mm. This technique led to a minimum error and variation between consecutive readings. The electronic gate contained within the FPGA acquisition card performed a running average of the last 32 waveforms on the signal to increase the signal-to-noise ratio and to enable more accurate tracking of the selected signal (see Figure 3c) by the gate.

**Figure 7:**
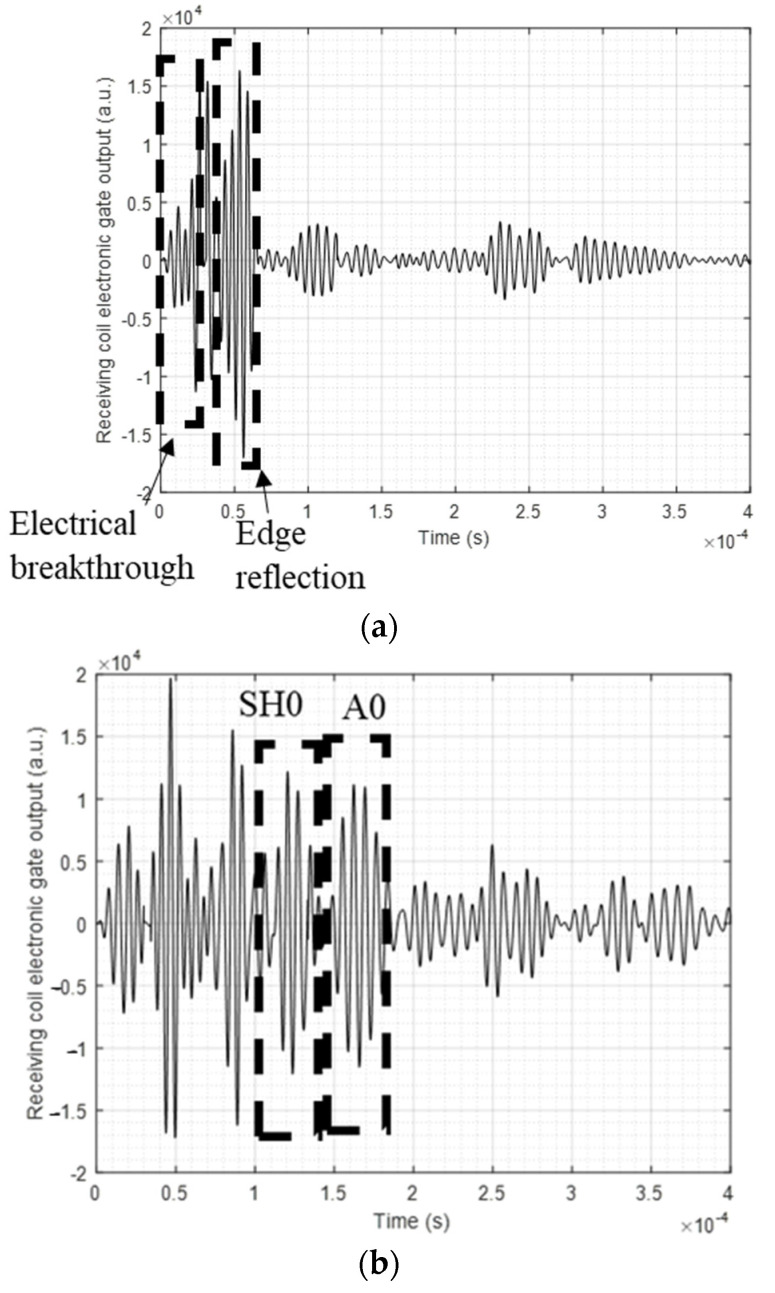
Collected signal from the Innerspec DAQ when the transducers and the sensor are placed (**a**) at the far point of the linear sweep (starting point, well away from the defect) and (**b**) directly opposite the defect location, showing SH0 and A0 reflected waves and the tracking gates used to select them. These data were collected using Configuration 1.

As reported in the previous section, a transmitter and receiver pair positioned a fixed distance apart performed a linear sweep on the composite plate, and different reflected signals were captured by the DAQ. The sweep was performed along the magnetostrictive patch strip, as shown earlier in Figure 3a. The electronic gate tracker was applied to differentiate between different arrivals captured by the detector, such as edge reflections and signals from the defect. A sample of a received time waveform when output from the electronic gate is shown in Figure 7. Figure 7a shows the signal with the transducer pair placed at a location well away from the defect (both using Configuration 1), where the signals were dominated by electrical feedthrough noise and edge reflections. Figure 7b then shows a signal where the transducer pair were directly opposite the defect, where the gated portions indicate the detection of A0 and SH0 signals arising from the defect. SH0 and A0 modes were identified by using the changes in arrival time with the position of the transducer pair along the magnetostrictive patch, and the gates applied accordingly. Note that, as expected, the A0 mode signal arrives after the SH0 mode signal because of its slower velocity.

Figure 7 indicates that, in addition to the expected guided wave modes, reflections from the sample edges were detected and identified using the known velocities in the sample. The gate output, which were applied as discussed earlier, allowed the detection and separation of different guided wave modes and their relative amplitude and time of arrival, aiding in the creation of SAFT-based images of defects. The different captured signals showed that the signal-to-noise ratio could be controlled and enhanced by switching between the different configurations and analyzing the different guided wave modes. In fact, using the same configuration and using the same inspection hardware, by only analyzing shear horizontal waves SH0 instead of antisymmetric modes A0, the SNR can be increased by around 8%, which leads to better defect imaging capabilities. Similarly, modifying the direction and amplitude of the applied dynamic and static magnetic fields (switching from a certain configuration to another) can greatly increase the detection capability and overall SNR.

**Figure 8:**
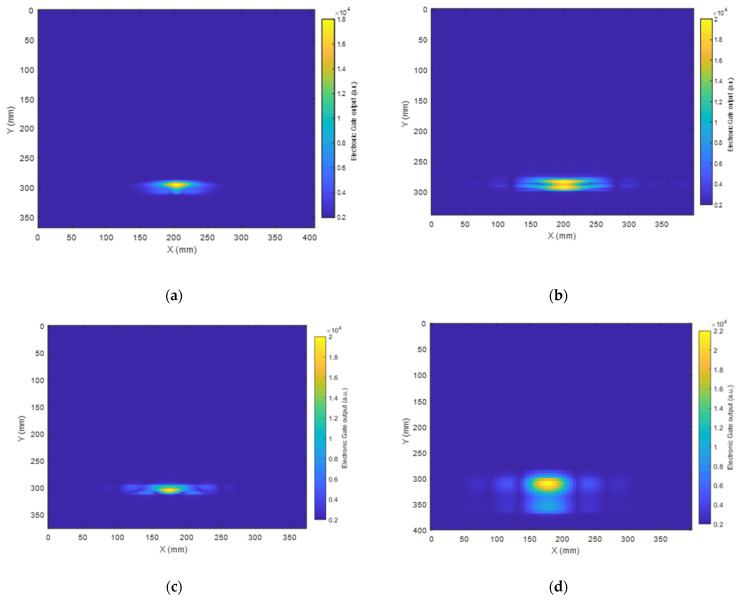
(**a**–**d**) SAFT images using the SH0 mode of the 25 mm wide circular artificial defect in the CFRP composite plate from Configurations 1–4, respectively.

**Figure 9:**
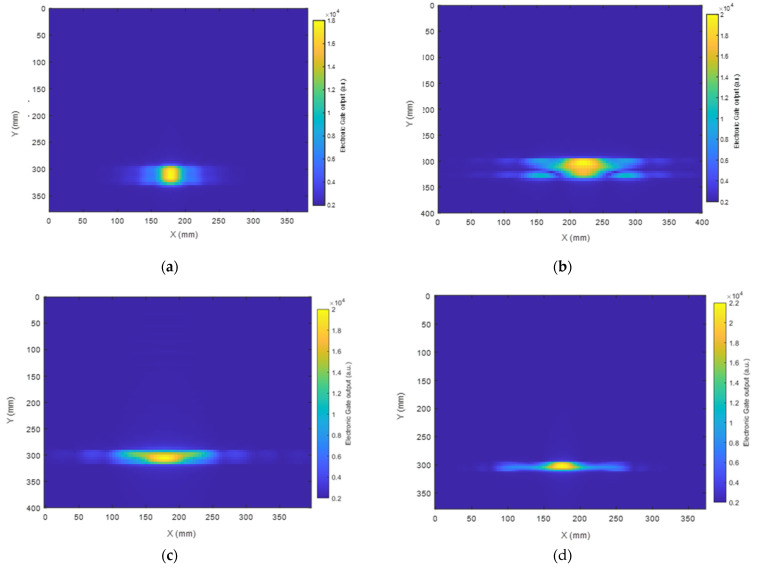
(**a**–**d**) SAFT images using the A0 mode of the artificial defect in the CFRP composite plate from Configurations 1–4, respectively.

The reconstructed images of the defect using the SAFT algorithm are presented in Figure 8 and Figure 9 using SH0 and A0 signals, respectively, as selected by the electronic gates in the Innerspec system with the know velocities of each mode at the center frequency of excitation. The spot in yellow marked in the images corresponds to the location where the reflected waves from the defect are at their maximum amplitude.

As presented in Figure 8, Configuration 1 led to the reconstruction of an image of the circular defect with a fairly accurate location, as the detection error was lower than 5% in terms of the defect location accuracy. Nevertheless, the shape of the defect was not defined fully. This is due to the fact that the detection window, as presented earlier, is narrow, which led to a limitation in data collection from the rear of the object. Configuration 2 clearly returned a more complex shape of the defect, while the location remained fairly accurate. Configuration 3, which exhibited a less directional radiation pattern, led to a more accurate estimate of the defect position in the X-Y plane. In fact, the wide generation and detection aperture provided by Configuration 3 allowed the capture of more relevant information on the area behind the defect with respect to the position of the transducer for this linear scan. Finally, Configuration 4 led to the reconstruction of a defect image with a fairly precise location with a clear shadow behind it.

An image of the defect was also constructed using the A0 mode and is presented in Figure 9. It can be seen that Configuration 1 led to the reconstruction of the defect image with an approximately circular outline of ~20 mm in diameter. Configuration 2 returned a more uniform shape of the defect compared than that produced using the SH0 mode (Figure 8b). It is also worth noticing that Configurations 2, 3, and 4 returned a more elliptical shape of the defect image.

### 4.3. Use of the SAFT Technique for Imaging an Artificial Delamination Defect in a GFRP Plate

**Figure 10:**
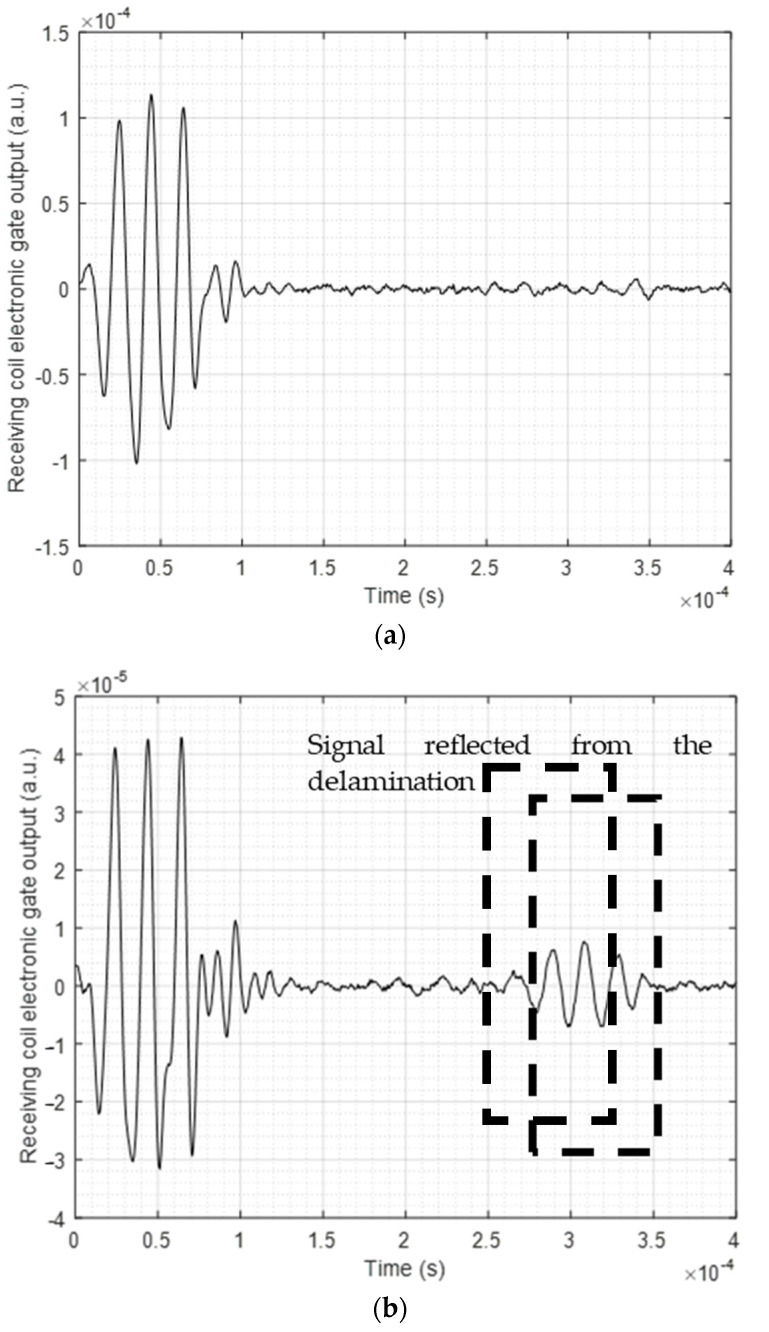
Collected signal (average of 32 points) showing the tracking gate set for the A0 mode when the transmitter and receiver pair were placed (**a**) in the absence of the defect (showing an electrical feedthrough noise signal only from the excitation waveform) and (**b**) in a region of the sample containing the delamination defect.

It can be seen in the above that the A0 mode yielded the best SAFT image reconstruction performance, and Configuration 1 particularly seemed to provide the best results in terms of image clarity. Consequently, Configuration 1 was used to generate A0-guided waves within a glass-fiber-reinforced polymer (GFRP) plate containing a 70 mm × 25 mm rectangular delamination defect in the form of an embedded PTFE film. An example of a received waveform is shown in Figure 10, where it can be seen that a clear defect reflection was captured.

**Figure 11:**
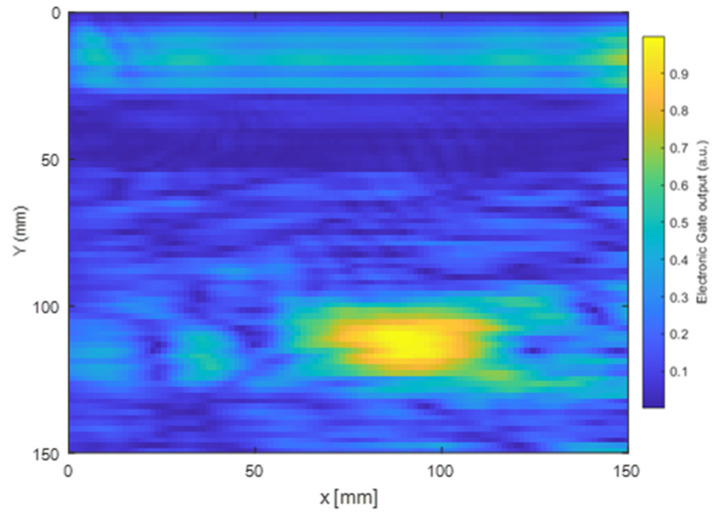
SAFT images of the delamination defect within the GFRP composite plate, reconstructed using the A0 mode and Configuration 1.

The result of SAFT imaging is shown in Figure 11, where the defect was successfully located. The size and location of the defect identified through the SAFT image were in good agreement with the known size and location of the defect. When inspecting the GFRP plate, the full signal was captured, as initially, the defect location was not identified. Conversely to the previous set of testing, the electronic gates were not fully used to suppress signal, which led to a difference in signal-to-noise ratio between both experiments.

## 5. Discussion and Conclusions

The use of Configuration 1 to provide the A0 Lamb wave mode was very successful in generating ultrasonic signals that had good properties for imaging, namely a reasonable directivity and good sensitivity. While it could also be used to generate the SH0 mode, the results of the SAFT imaging were not as good as that obtained with the A0 mode. In fact, the amplitude of the reflected signal received from the defect increased by 15% when the A0 wave mode was used to reconstruct the defect image compared to the use of SH0 in the case of configuration 1. The location of the defect was identified with high accuracy as the detection error was lower than 5% for both situations where the defect image was reconstructed using SH0 waves and A0 mode waves. This could be due to the nature of the defects; the SH0 would not be particularly sensitive to delamination defects in any case, and hence the A0 mode would normally be preferred. Other configurations did result in SAFT images, but Configuration 1 and the A0 mode seemed to be the best approach. A careful selection of the mode and the different parameters is essential to reconstruct the best image possible of the defect. Although the amplitude of the A0 signal reflected from the defect using configuration 1 is lower than the other configurations by around 10%, the imaging capability of this configuration remained the highest. Note, though, that when Configuration 2 was used, the defect was detectable from further distances when compared to configuration 1. In fact, the distance of the defect detection increased by 25% when configuration 2 is used instead of configuration 1 while performing the linear sweep. This was due to the fact that this configuration provided a wider beam aperture, which led to a better detection capability over a wider range of angles.

The use of configuration 3 led to the generation of elastic waves with a less directional radiation pattern. This radiation pattern allowed the detection of the defect across a wider aperture (i.e., greater lateral distances from the defect) compared to configurations 1 and 2. This allowed Configuration 3 to capture key elements in the region behind the defect. It is interesting to note that using the SH0 mode with Configuration 3 led to an elliptically shaped image rather than its known circular shape. This effect was also present when the A0 mode was used, but with the A0 mode, the resolution in the Y direction was enhanced. When the A0 wave mode was used, the region behind the defect was properly captured, leading to a significant reduction in noise.

The use of Configuration 4 resulted in a wide beam aperture for both A0 and SH0 modes when generated in the CFRP composite plate. Using this configuration in conjunction with the SH0 mode led to the reconstruction of the defect image with reasonably good results in both the X and Y directions. This is due to the fact that the generated SH0 beam was relatively narrow, which led to reduced capability to detect the area behind the defect. The amplitude of the reflected signal using SH0 wave mode in configuration 4 was increased by 10% compared to the signal obtained by analyzing the A0 wave mode.

The results indicated that different configurations were capable of producing different radiated field characteristics and hence different image reconstruction features using the orientation of the applied static and dynamic magnetic fields (*B_s_* and *B_d_*, respectively). The detection capability and the location of the defect within the plate were considered to be of fairly good accuracy as the error margin was lower than 5% in all configurations when using either the A0 waves or SH0 mode. The accuracy of detection was enhanced by modifying the direction and amplitude of the static magnetic field and the dynamic magnetic field. The nature of the generation of all three guided modes investigated here (SH0, A0, and S0) could be adjusted, but it was found that the S0 was not favored as greatly as SH0 and A0 generation, with the latter giving the best results in the samples investigated. In fact, the S0 mode amplitude was relatively low compared to the SH0 and A0 modes, which led to high attenuation levels and challenges in distinguishing different reflected wave modes in order to detect the defect.

The second set of experiments on the GFRP plate containing a delamination defect led to a good detection sensitivity when Configuration 1 and the A0 mode were used. Note that this is to be expected due to the large out-of-plane motion of this mode. As explained earlier, the SH0 mode signals are not expected to be as useful for such a measurement due to their in-plane motion.

Finally, a series of experiments were performed in order to evaluate the use of magnetostrictive patches adhered to the surface of composite samples as a method for imaging defects using SAFT reconstruction techniques. A range of static and magnetic field configurations was studied, and it was found that optimum signals for image reconstruction resulted from the use of Configuration 1 to generate and detect A0 wave modes of detecting simulated defects in the composite samples used. These modes were used to reconstruct good images of artificial defects in both CFRP and GFRP plates.

## Figures and Tables

**Table 1:** *B_s_* and *B_d_* directions and coil shapes used in each configuration.

Configuration	*B_s_* Direction	*B_d_* Direction	Coil Shape
Configuration 1	*x*–axis	*y*–axis	Racetrack
Configuration 2	*x*–axis	*x*–axis	Racetrack
Configuration 3	*z*–axis	Radial (X, Y plane)	Circular
Configuration 4	*x*–axis	Radial (X, Y plane)	Circular

**Table 2:** Computed phase and group velocity for ultrasonic guided waves at 120 kHz generated in a 3 mm CFRP plate.

Guided Waves Mode	Phase Velocity (m/s)	Group Velocity (m/s)
A0	1413	1738
S0	6738	6719
SH0	2034	2034

**Table 3:** Mechanical and magnetic characteristics of the magnetostrictive patch material (VACOFLUX iron-cobalt alloy).

Young’s Modulus	200 GPa
Poisson ratio	0.29
Density	8.12 g/cm^3^
Electrical resistivity	0.42 µΩm
Saturation magnetostriction	70 ppm
Saturation magnetization	2.35 T

## Data Availability

Not applicable.
